# A Facile Synthesis and Antimicrobial Activity Evaluation of Sydnonyl-Substituted Thiazolidine Derivatives

**DOI:** 10.3390/molecules20046520

**Published:** 2015-04-13

**Authors:** Mei-Hsiu Shih, Yu-Yuan Xu, Yu-Sheng Yang, Guan-Ling Lin

**Affiliations:** Department of Chemical and Materials Engineering, Southern Taiwan University of Science and Technology, Tainan City 71005, Taiwan; E-Mails: ma140107@stust.edu.tw (Y.-Y.X.); ma140201@stust.edu.tw (Y.-S.Y.); lynnlin0221@yahoo.com.tw (G.-L.L.)

**Keywords:** Knoevenagel reaction, sydnones, thiazolidines, antimicrobial activity

## Abstract

Some new sydnonyl-substituted thiazolidine derivatives were synthesized in high yields by the modified Knoevenagel condensation of 3-aryl-4-formylsydnones with thiazolidine-2,4-dione and 2-thioxo-thiazolidine-4-one, respectively. All the synthesized thiazolidine derivatives were screened by paper-disc method to identify their antimicrobial activities against three bacteria viz. *Staphylococcus aureus*, *Proteus vulgaris* and *Escherichia coli*, and two fungal cultures viz. *Aspergillus niger* and *Penicillium citrinum*. The reference drugs were Norfloxacin and Griseofulvin, respectively. The screening data indicated that the tested sydnonyl-substituted thiazolidine derivatives exhibited no obvious antibacterial activity compared with the standard drug Norfloxacin. However, thiazolidine derivatives displayed significant antifungal activities against *Penicillium citrinum* and *Aspergillus niger*. Notably, all of the tested compounds showed growth inhibitory activity 1.5-4.4 times higher than that of the standard drug Griseofulvin against the two fungi.

## 1. Introduction

Thiazolidines, thiazolidinones and their derivatives have attracted continuing interest over the years because of their varied biological activities, such as antitumor [1,2,3], anticancer [4,5,6], anti-inflammatory [7], antimicrobial [8,9,10] and anti-*Toxoplasma gondii* activities [11]. Consequently, chemists still enthusiastically pursue the syntheses and activity evaluation of thiazolidine or thiazolidinone derivatives [12,13,14,15,16]. 3-Aryl-4-formylsydnones **1** have extensively been studied since their discovery [17] and their applications have been investigated [18,19,20,21,22,23,24]. Several sydnone derivatives are also associated with a wide range of pharmacological activities, exhibiting antimicrobial, anti-inflammatory, antioxidant, antitumor and anticancer properties [25,26,27,28]. Thus, syntheses of sydnone derivatives substituted with thiazolidine or thiazolidinone group at a suitable position by a convenient method is an important part of developing new and potentially biologically active compounds. In view of the continued interest in the development of simpler and more convenient synthetic routes for preparing sydnonyl-substituted heterocyclic systems, an efficient and useful method is reported herein to synthesize some new sydnonyl-substituted thiazolidinone derivatives by the modified Knoevenagel condensation of 3-aryl-4-formylsydnones **1a**–**d** with thiazolidine-2,4-dione (**2**) and 2-thioxo-thiazolidin-4-one (**3**), respectively. All the synthesized thiazolidinone derivatives **4a**–**d**, **5a**–**d** were screened by paper-disc method to identify their antimicrobial activities against three bacteria viz. *Staphylococcus aureus, Proteus vulgaris* and *Escherichia coli*, and two fungal cultures viz. *Aspergillus niger* and *Penicillium citrinum*.

## 2. Results and Discussion

### 2.1. Synthetic Chemistry

Knoevenagel condensation is now a very established method for the synthesis of α,β-unsaturated carbonyl compounds by condensation of aldehydes or ketones with C-H acidic methylene group-containing compounds. In general, this condensation is usually carried out homogeneously using bases such as ammonia and ammonium salts, aliphatic amines and their salts as catalysts [29,30,31,32]. It can also be performed in the heterogeneous phase using an inorganic catalyst such as titanium tetrachloride, tellurium tetrachloride or aluminum oxide [33,34,35].

**Scheme 1:**
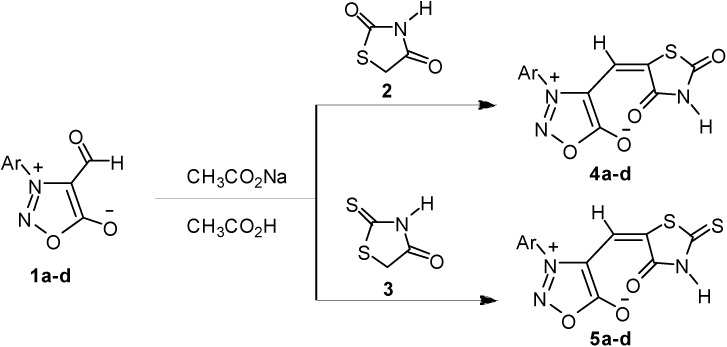
Synthesis of thiazolidinone derivatives **4a**–**d**, **5a**–**d** from sydnone compounds **1a**–**d**. **a**: Ar = C_6_H_5_; **b**: Ar = *p*-CH_3_C_6_H_4_; **c**: Ar = *p*-CH_3_OC_6_H_4_; **d**: Ar = *p*-C_2_H_5_OC_6_H_4_.

In this work, 3-aryl-4-formylsydnones **1** were at first treated with active methylene compounds, thiazolidine-2,4-dione (**2**) or 2-thioxo-thiazolidin-4-one (**3**) in piperidine/glacial acetic acid buffer system. However, the sydnone ring itself is sensitive to acids, bases and heat. Sydnone compounds are sometimes decomposed during reaction and/or work-up. Hence, the Knoevenagel condensations were controlled at low temperature by successive addition of glacial acetic acid, methylene compounds with piperidine to the sydnone ethanol solution in order. However, the starting materials **1** were still unavoidably decomposed. Several tests established glacial acetic acid was first added to the ice-cooled ethanol solution of sydnone **1** since sydnone compound was absolutely not decomposed in acetic acid. Then, the ice-cooled ethanol solution of the active methylene compound with sodium acetate was slowly added to the above acidic solution and the mixture was stirred to precipitate out the solid. We also detected two or more products in the reaction mixture. Finally, directly using glacial acetic acid as solvent rather than ethanol improved the reaction result, we found only one product in the reaction by TLC detection. Consequently, under optimal experimental condition, starting materials **1a**–**d** reacted with activated methylene compound thiazolidine-2,4-dione (**2**) at 80 °C in glacial acetic acid/sodium acetate buffer system to give condensation products **4a**–**d** successfully. Compounds **5a**–**d** were obtained by the reaction of 2-thioxo-thiazolidin-4-one (**3**) with starting materials **1a**–**d** through the same reaction condition mentioned above (Scheme 1). The pH value about 4.7 of glacial acetic acid/sodium acetate buffer system was very suitable for sydnone derivative syntheses and the sydnone ring was absolutely not decomposed.

**Figure 1:**
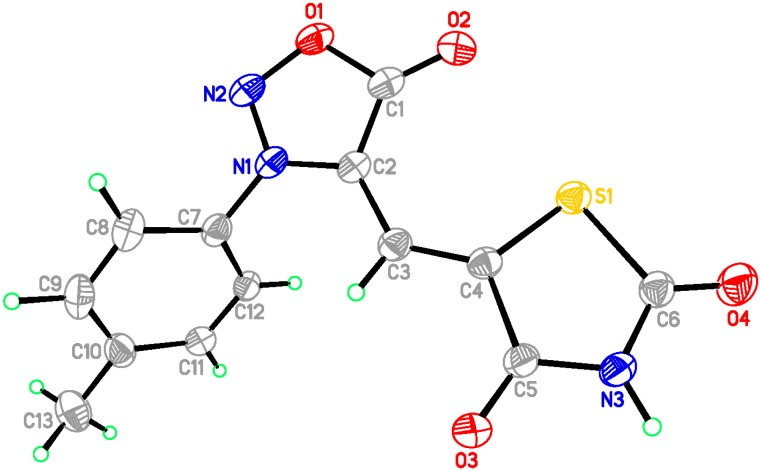
ORTEP drawing of 5-[3-(4-methylphenyl)sydnon-4-ylmethylene]thiazolidine-2,4-dione (**4b**).

**Figure 2:**
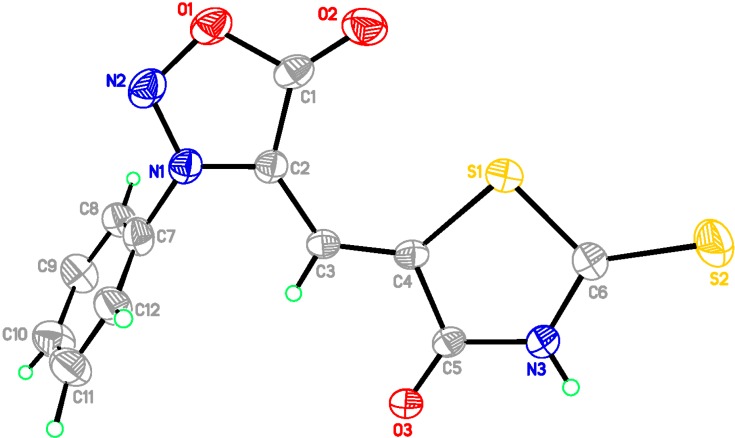
ORTEP drawing of 5-(3-phenylsydnon-4-ylmethylene)-2-thioxothiazolidin-4-one (**5a**).

**Figure 3:**
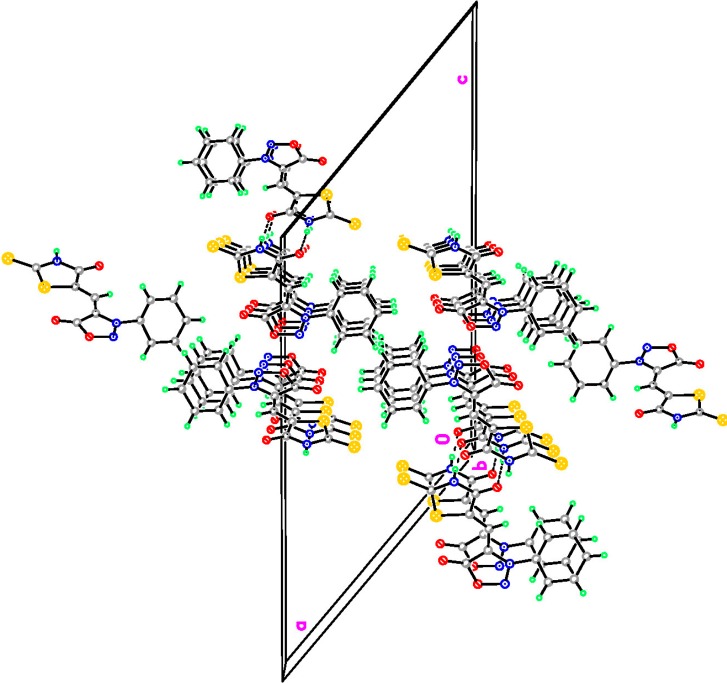
View along the b axis of packing diagram of compound **5a**.

All these synthesized products were spectrally characterized by IR, ^1^H-NMR, ^13^C-NMR, MS and Elemental Analyses. Among these new compounds **4a**–**4d**, **5a**–**5d**, the crystals **4b**, and **5a** were also analyzed by X-ray diffraction. Figure 1 and Figure 2 show the molecular structures of compounds **4b** and **5a**. Details of the crystal data of compounds **4b** and **5a** are given in Table 1. Moreover, view along the b axis of packing diagram of compound **5a** is displayed in Figure 3.

**Table 1:** Crystal Data of compounds **4a** and **5b**.

Compounds	4b	5a
Diffractometer	Nonius Kappa CCD	Nonius Kappa CCD
Formula	C_1__3_H_9_N_3_O_4_S	C_1__2_H_7_N_3_O_3_S_2_
Formula weight	303.29	305.33
Crystal system	Monoclinic	Monoclinic
Space group	P2(1)/c	P2(1)/c
*a*/Å	7.46360(10)	16.749(3)
*b*/Å	22.0840(5)	4.9332(10)
*c*/Å	8.2955(2)	24.777(5)
*α*/°	90.00	90.00
*β*/°	98.2900(14)	140.16(3)
*γ*/°	90.00	90.00
*V*/Å^3^	1353.03(5)	1311.7(5)
*Z*	4	4
*D*_calc_ (g·cm^−3^)	1.489	1.546
*F*_000_	624.00	624
Absorption coefficient (mm^−1^)	0.259	0.416
Crystal size/mm	0.30 × 0.25 × 0.20	0.30 × 0.25 × 0.20
Temperature (K)	295(2)	295(2)
θ_range_, deg	1.84–27.47	1.65–27.49
Reflections collected	8949	13460
Independent reflections	3075 [ *R*(_int_) = 0.0318]	2940 [ *R*(_int_) = 0.1322]
Refinement method	Full-matrix least-squares on *F*^2^	Full-matrix least-squares on *F*^2^
Final *R* indices [*I* > 2.00σ(*I*)]	*R*_1_ = 0.0427, *_W_R*_2_ = 0.1123	*R*_1_ = 0.0585, *_W_R*_2_ = 0.1584
*R* indices (all data)	*R*_1_ = 0.0653, *_W_R*_2_ = 0.1339	*R*_1_ = 0.0814, *_W_R*_2_ = 0.1904
GoF	1.096	1.052

### 2.2. Evaluation of Antimicrobial Activity 

All these compounds were screened to determine their antimicrobial activity *in vitro* against three bacteria viz. *Staphylococcus aureus, Proteus vulgaris* and *Escherichia coli*, and two fungal cultures viz. *Aspergillus niger* and *Penicillium citrinum*. The reference drugs were Norfloxacin (N) for antibacterial and Griseofulvin (G) for antifungal tests, respectively. The tests were performed with the title compounds and the reference drugs, under identical conditions by the paper-disc method using the adequate quantity (30 μg) of the substance in 50 μL of DMF. The total inhibition area was calculated by the inhibition zone, in comparison with the reference drug, as follows: Relative inhibition % = 100(*X* − *Y*)/(*Z* − *Y*); *X* = total inhibition area in the test compound; *Y* = total inhibition area in DMF; *Z* = total inhibition area in the reference drug [36,37].

**Figure 4:**
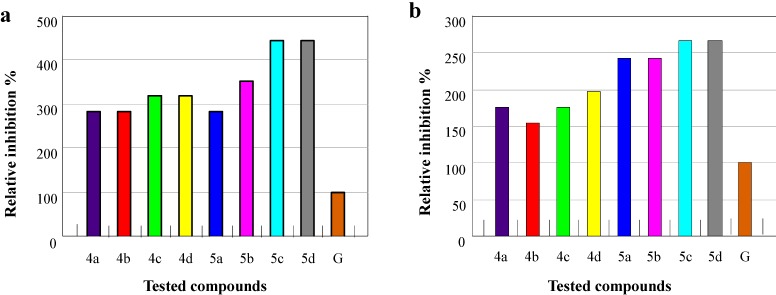
(**a**) The relative inhibition activity of compounds **4a**–**d** and **5a**–**d** against *Aspergillus niger*; (**b**) The relative inhibition activity of compounds **4a**–**d** and **5a**–**d** against *Penicillium citrinum*. G: Griseofulvin.

The screening data indicated that the tested compounds **4a**–**d** and **5a**–**d** did not show very obvious degree of antibacterial activities against *Staphylococcus aureus*, *Proteus vulgaris* and *Escherichia coli*, compared with the standard drug Norfloxacin. The growth inhibitory activities of all test compounds are lower than that of the standard drug Norfloxacin against three bacteria, and the relative Inhibition (%) are about 11%–77%. However, compounds **4a**–**d** and **5a**–**d** displayed significant antifungal activities against both fungi *Aspergillus niger* and *Penicillium citrinum*, as listed in Table 2. Notably, all of the tested compounds showed growth inhibitory activity 1.5–4.4 times higher than that of the standard drug Griseofulvin against the two fungal cultures*.* Among these thiazolidine derivatives, compounds **5a**–**d** carrying 2-thioxothiazolidin-4-one group displayed better antimicrobial activity against both fungi than thiazolidine-2,4-diones **4a**–**d** did. Especially, compounds **5c** and **5d** with 3-(4-methoxyphenyl)sydnon-4-yl and 3-(4-ethoxyphenyl)sydnon-4-yl moiety, respectively, exhibited the best antimicrobial activity against both fungi, *Aspergillus niger* and *Penicillium citrinum*, as shown in Table 2 and Figure 4. The study of the syntheses of new sydnonyl-substituted thiazolidines with antimicrobial activity might support the development of new drugs and improve the treatment of infectious diseases.

**Table 2:** Antifungal activity of sydnonyl-substituted thiazolidine derivatives **4a**–**d**, **5a**–**d**.

Compounds	*Aspergillus Niger* *		*Penicillum Citrinum* *	
	Inhibition Zone (mm)	Relative Inhibition (%)	Inhibition Zone (mm)	Relative Inhibition (%)
**4a** (Ar = C_6_H_5_)	19	282.88 ± 5.91	20	175.01 ± 3.81
**4b** (Ar = *p*-CH_3_C_6_H_4_)	19	282.88 ± 6.61	19	154.70 ± 1.62
**4c** (Ar = *p*-CH_3_OC_6_H_4_)	20	320.04 ± 9.32	20	175.07 ± 8.51
**4d** (Ar = *p*-C_2_H_5_OC_6_H_4_)	20	320.06 ± 11.21	21	196.38 ± 5.05
**5a** (Ar = C_6_H_5_)	19	282.91 ± 9.34	23	242.24 ± 6.51
**5b** (Ar = *p*-CH_3_C_6_H_4_)	21	351.11 ± 7.93	23	242.23 ± 7.59
**5c** (Ar = *p*-CH_3_OC_6_H_4_)	23	442.92 ± 9.39	24	266.68 ± 4.56
**5d** (Ar = *p*-C_2_H_5_OC_6_H_4_)	23	442.87 ± 12.37	24	266.67 ± 2.89
**G** (Griseofulvin)	13	100	16	100
**B** (DMF)	8	**-**	8	**-**

* Concentration of tested compounds in the antifungal activity: 30 μg/50 μL.

## 3. Experimental Section

### 3.1. General

All melting points were determined on an England Electrothermal Digital Melting Point apparatus and were uncorrected. IR spectra were recorded on a MATTSON/SATELLITE 5000 FT-IR spectrophotometer (Madison, WI, USA). Mass spectra were measured on a high-resolution mass spectrometer JEOL JMS-700 (Tokyo, Japan) and Bruker FT-MS APEX II (Rheinstetten, Germany). ^1^H-NMR spectra were run on a Bruker AV 400 NMR spectrometer (Rheinstetten, Germany), using TMS as an internal standard. ^13^C-NMR spectra were carried out with complete ^1^H decoupling and assignments were made through additional DEPT experiments. Elemental analyses were taken with an Elementar Vario EL-III Analyzer (Hanau, Germany). X-ray spectra were performed on *Nonious* Kappa CCD Single-crystal XRD (Bruker, Germany). 3-Aryl-4-formylsydnones (**1a**–**d**) were prepared from the corresponding 3-arylsydnones according to the literature [17]. 

### 3.2. Syntheses of 5-(3-Arylsydnon-4-ylmethylene)thiazolidine-2,4-diones **4a**–**d**

To a solution of thiazolidine-2,4-dione (**2**, 176.0 mg, 1.5 mmol) and sodium acetate (246.1 mg, 3.0 mmol) in 3 mL of glacial acetic acid, 3-phenyl-4-formylsydnone (1**a**, 190.2 mg, 1.0 mmol) was slowly added at room temperature. The mixed solution was heated at 80 °C for 1–3 days until the reaction was completed and then cooled. The precipitating yellow powder (216.2 mg) was collected by filtration and washed with ice-cold water, cold ethanol, and after recrystallization from acetone-ethanol afford 199.8 mg of 5-(3-phenylsydnon-4-ylmethylene)thiazolidine-2,4-dione (**4a**) as yellow needles, yield 69%. The chemical and physical spectral characteristics of these products **4a**–**d** are given below.

*5-(3-Phenylsydnon-4-ylmethylene)thiazolidine-2,4-dione* (**4a**): Yellow needles from CH_3_COCH_3_/EtOH; yield 69%; mp 260–261 °C; IR (KBr) 3144, 3039, 2762, 1744, 1678, 1598, 1323, 1290, 1012, 769, 552 cm^−1^; ^1^H-NMR (DMSO-*d*_6_) δ 6.89 (s, 1H, HC=C), 7.72–7.87 (m, 5H, ArH), 12.53 (s, 1H, NH); ^13^C-NMR (DMSO-*d*_6_) δ 107.01, 113.86, 122.25, 125.95, 130.63, 132.71, 133.28, 164.71, 167.31, 168.61; FABMS *m/z* (%): 290 ([M+H]^+^, 100), 289 (M^+^, 91); Anal. Calcd for C_12_H_7_N_3_O_4_S: C, 49.83; H, 2.44; N, 14.53. Found: C, 49.94; H, 2.56; N, 14.55. 

*5*-[3-(4-Methylphenyl)sydnon-4-ylmethylene]*thiazolidine-2,4-dione* (**4b**): Yellow crystals from CH_3_COCH_3_/EtOH; yield 77%; mp 239–240 °C; IR (KBr) 3126, 3037, 2791, 1749, 1692, 1600, 1332, 1277, 1174, 826, 612 cm^−1^; ^1^H-NMR (DMSO-*d*_6_) δ 2.52 (s, 3H, CH_3_), 6.92 (s, 1H, HC=C), 7.60 (d, *J* = 7.6 Hz, 2H, ArH), 7.73 (d, *J* = 7.6 Hz, 2H, ArH), 12.53 (s, 1H, NH); ^13^C-NMR (DMSO-*d*_6_) δ 21.12, 106.95, 113.96, 122.11, 125.64, 130.23, 130.95, 143.67, 164.75, 167.36, 168.63; FABMS *m/z* (%): 304 ([M+H]^+^, 100), 303 (M^+^, 38); Anal. Calcd for C_13_H_9_N_3_O_4_S: C, 51.48; H, 2.99; N, 13.85. Found: C, 51.45; H, 3.01; N, 13.82. X-ray analytical data is listed in Table 1. Further details have been deposited at the Cambridge Crystallographic Data Center and allocated the deposition number CCDC 1048337.

*5*-[3-(4-Methoxyphenyl)sydnon-4-ylmethylene]*thiazolidine-2,4-dione* (**4c**): Yellow powder from CH_3_COCH_3_/EtOH; yield 67%; mp 225–226 °C; IR (KBr) 3157, 3067, 2769, 1754, 1717, 1607, 1510, 1319, 1262, 1171, 836, 610 cm^−1^; ^1^H-NMR (DMSO-*d*_6_) δ 3.89 (s, 3H, CH_3_O), 6.91 (s, 1H, HC=C), 7.29 (d, *J* = 9.2 Hz, 2H, ArH), 7.76 (d, *J* = 9.2 Hz, 2H, ArH), 12.50 (s, 1H, NH); ^13^C-NMR (DMSO-*d*_6_) δ 56.11, 107.04, 114.15, 115.63, 121.90, 125.17, 127.47, 162.49, 164.78, 167.35, 168.64; FABMS *m/z* (%): 320 ([M+H]^+^, 100), 319 (M^+^, 38); Anal. Calcd for C_13_H_9_N_3_O_5_S: C, 48.90; H, 2.84; N, 13.16. Found: C, 48.74; H, 2.92; N, 13.05.

*5*-[3-(4-Ethoxyphenyl)sydnon-4-ylmethylene]*thiazolidine-2,4-dione* (**4d**): Yellow needles from CH_3_COCH_3_/EtOH; yield 67%; mp 219–220 °C; IR (KBr) 3176, 3074, 2761, 1759, 1722, 1605, 1509, 1318, 1260, 1173, 844, 610 cm^−1^; ^1^H-NMR (DMSO-*d*_6_) δ 1.37 (t, *J* = 6.8 Hz, 3H, CH_3_), 4.17 (q, *J* = 6.8 Hz, 2H, CH_2_), 6.91 (s, 1H, HC=C), 7.26 (d, *J* = 8.8Hz, 2H, ArH), 7.74 (d, *J* = 8.8Hz, 2H, ArH), 12.50 (s, 1H, NH); ^13^C-NMR (DMSO-*d*_6_) δ 14.60, 64.20, 107.02, 114.16, 115.94, 121.85, 124.99, 127.45, 161.78, 164.77, 167.32, 168.63; FABMS *m/z* (%): 334 ([M+H]^+^, 100), 333 (M^+^, 35); Anal. Calcd for C_14_H_11_N_3_O_5_S: C, 50.45; H, 3.33; N, 12.61. Found: C, 50.32; H, 3.32; N, 12.45.

### 3.3. Syntheses of 5-(3-Arylsydnon-4-ylmethylene)-2-thioxothiazolidin-4-ones **5a**–**d**

To a solution of 2-thioxo-4-thiazolidinone (**3**, 199.8 mg, 1.5 mmol) and sodium acetate (246.1 mg, 3.0 mmol) in 3 mL of glacial acetic acid, 3-phenyl-4-formylsydnone (**1a**, 190.2 mg, 1.0 mmol) was slowly added at room temperature. The mixed solution was heated at 80 °C for 1–2 d until the reaction was completed and then cooled. The precipitating orange red powder (295.5 mg) was collected by filtration and washed with ice-cold water, cold ethanol, and after recrystallization from acetone-ethanol afford 268.8 mg of 5-(3-phenylsydnon-4-ylmethylene)-2-thioxothiazolidin-4-one (**5a**) as orange red needles, yield 88%. The chemical and physical spectral characteristics of these products **5a**–**d** are given below.

*5-(3-Phenylsydnon-4-ylmethylene)-2-thioxothiazolidin-4-one* (**5a**): Orange red needles from CH_3_COCH_3_/EtOH; yield 88%; mp 243–244 °C; IR (KBr) 3134, 3036, 2843, 1761, 1683, 1578, 1314, 1215, 1077, 768, 589 cm^−1^; ^1^H-NMR (DMSO-*d*_6_) δ 6.71 (s, 1H, HC=C), 7.64–7.92 (m, 5H, ArH), 13.72 (s, 1H, NH); ^13^C-NMR (DMSO-*d*_6_) δ 107.71, 113.18, 123.97, 125.95, 130.66, 132.59, 133.36, 164.87, 169.40, 196.43; FABMS *m/z* (%): 306 ([M+H]^+^, 100), 305 (M^+^, 26); Anal. Calcd for C_12_H_7_N_3_O_3_S_2_: C, 47.20; H, 2.31; N, 13.76. Found: C, 47.35; H, 2.45; N, 13.71. X-ray analytical data is listed in Table 1. Further details have been deposited at the Cambridge Crystallographic Data Center (www.ccdc.cam.ac.uk/conts/retrieving.html) and allocated the deposition number CCDC 1048338.

5-[3-(4-Methylphenyl)sydnon-4-ylmethylene]-*2-thioxothiazolidin-4-one* (**5b**): Orange red crystals from CH_3_COCH_3_/EtOH; yield 79%; mp 245–246 °C; IR (KBr) 3149, 3048, 2867, 1743, 1690, 1587, 1318, 1225, 1178, 819, 679 cm^−1^; ^1^H-NMR (DMSO-*d*_6_) δ 2.51 (s, 3H, CH_3_), 6.74 (s, 1H, HC=C), 7.61 (d, *J* = 8.0 Hz, 2H, ArH), 7.74 (d, *J* = 8.0 Hz, 2H, ArH), 13.74 (s, 1H, NH); ^13^C-NMR (DMSO-*d*_6_) δ 21.14, 107.60, 113.34, 123.79, 125.66, 130.12, 131.00, 143.78, 164.88, 169.40, 196.43; FABMS *m/z* (%): 320 ([M+H]^+^, 100), 319 (M^+^, 33); Anal. Calcd for C_13_H_9_N_3_O_3_S_2_: C, 48.89; H, 2.84; N, 13.16. Found: C, 49.13; H, 3.00; N, 12.76. 

5-[3-(4-Methoxyphenyl)sydnon-4-ylmethylene]-*2-thioxothiazolidin-4-one* (**5c**): Orange red needles from CH_3_COCH_3_/EtOH; yield 85%; mp 230–231 °C; IR (KBr) 3154, 3004, 2962, 1770, 1683, 1579, 1428, 1311, 1268, 1178, 839, 679 cm^−1^; ^1^H-NMR (DMSO-*d*_6_) δ 3.90 (s, 3H CH_3_O), 6.74 (s, 1H, HC=C), 7.30 (d, *J* = 8.8 Hz, 2H, ArH), 7.77 (d, *J* = 8.8 Hz, 2H, ArH), 13.70 (s, 1H, NH); ^13^C-NMR (DMSO-*d*_6_) δ 56.16, 107.71, 113.53, 115.71, 123.66, 125.07, 127.50, 162.58, 164.92, 169.45, 196.48; FABMS *m/z* (%): 336 ([M+H]^+^, 100), 335 (M^+^, 37); Anal. Calcd for C_13_H_9_N_3_O_4_S_2_: C, 46.56; H, 2.71; N, 12.53. Found: C, 46.72; H, 2.75; N, 12.45.

5-[3-(4-Ethoxyphenyl)sydnon-4-ylmethylene]-*2-thioxothiazolidin-4-one* (**5d**): Orange red needles from CH_3_COCH_3_/EtOH; yield 87%; mp 244–245 °C; IR (KBr) 3134, 3041, 2848, 1759, 1682, 1568, 1508, 1312, 1256, 1225, 839, 682 cm^−1^; ^1^H-NMR (DMSO-*d*_6_) δ 1.37 (t, *J* = 7.2 Hz, 3H, CH_3_), 4.17(q, *J* = 7.2 Hz, 2H, CH_2_O), 6.73 (s, 1H, HC=C), 7.27 (d, *J* = 9.2 Hz, 2H, ArH), 7.75(d, *J* = 9.2 Hz, 2H, ArH), 13.71 (s, 1H, NH); ^13^C-NMR (DMSO-*d*_6_) δ 14.60, 54.23, 107.68, 113.53, 116.00, 123.58, 124.87, 127.48, 161.87, 164.90, 169.40, 196.44; FABMS *m/z* (%): 350 ([M+H]^+^, 100), 349 (M^+^, 35); Anal. Calcd for C_14_H_11_N_3_O_4_S_2_: C, 48.13; H, 3.17; N, 12.03. Found: C, 48.24; H, 3.23; N, 11.91. 

### 3.4. Biological Evaluation (Antimicrobial Activity) 

The antimicrobial activities of synthesized compounds **4a**–**d** and **5a**–**d** were investigated *in vitro* using five microorganisms. These organisms included three bacteria (1, *Staphylococcus aureus* ATCC-12600; 2, *Proteus vulgaris* ATCC-13315; 3, *Escherichia coli* CCRC-10316), and two fungi (4, *Aspergillus niger* ATCC-42418 and 5, *Penicillium citrinum*. ATCC-8506). The microorganisms were provided by the Culture Collection and Research Center of FIRDI in Taiwan and the American Type Culture Collection, Manassas, VA, USA. The tests were carried out with the synthesized compounds **4a**–**d** and **5a**–**d** and the reference drugs, under identical conditions using the paper-disc method with the adequate quantity (30 μg) of the substance in 50 μL of DMF. The reference drugs used were Norfloxacin for antibacterial activities and Griseofulvin for antifungal activity, respectively. First, the testing plates with double layer agar were prepared. The base layer contains nutrient agar for bacteria and potato dextrose agar for fungi, respectively. Meanwhile, the upper layer comprises water agar containing bacteria or fungi. The discs of the filter paper (8 mm diameter) were placed in a petri dish and sterilized at 125 °C for 2 h. Following cooling, 50 μL of the compound solution was added to each paper-disc. After drying in the lamilaflow, the paper-disc containing test compound was placed on a petri dish with a double layer of nutrient agar and water agar. The plates were incubated at a suitable temperature (37 °C for bacteria, 26 °C for fungi) for 1–4 days. The inhibition zones were observed and determined. All the tests were undertaken on four replicates and the results were averaged. The total inhibition area was calculated using the inhibition zone, in comparison with the reference drug, as follows: Relative inhibition % = 100(*X* − *Y*)/(*Z* − *Y*); *X* = total area of inhibition in the test compound; *Y* = total area of inhibition in DMF; *Z* = total area of inhibition in the reference drug [36,37].

## 4. Conclusions

For detailed study of biological activity, some new sydnonyl-substituted thiazolidine derivatives were successfully synthesized by the modified Knoevenagel condensation of 3-aryl-4-formylsydnones with thiazolidine-2,4-dione and 2-thioxo-thiazolidin-4-one respectively. The evaluation of antimicrobial activity indicated that the synthesized compounds **4a**–**d** an**d5a**–**d** showed less antibacterial activities against *Staphylococcus aureus*, *Proteus vulgaris* and *Escherichia coli*, comparable to the standard drug Norfloxacin. However, compounds **4a**–**d** and **5a**–**d** showed growth inhibitory activity 1.5–4.4 times higher than that of the standard drug Griseofulvin against *Penicillium citrinum*and*Aspergillus niger*. Among these thiazolidinone derivatives, compounds **5a**–**d** carrying 2-thioxo-thiazolidin-4-one group displayed better antimicrobial activity against both fungi than thiazolidine-2,4-diones **4a**–**d** did. Especially, substituents 4-methoxy and 4-ethoxy groups on the 3-arylsydnone ring of compounds **5c** and **5d** respectively, increased the antifungal activity compared with compounds **5a** and **5b** against both of fungi, *Aspergillus niger* and *Penicillium citrinum.*
